# Recommendations for Services in a FAIR Data Ecosystem

**DOI:** 10.1016/j.patter.2020.100104

**Published:** 2020-09-11

**Authors:** Hylke Koers, Daniel Bangert, Emilie Hermans, René van Horik, Maaike de Jong, Mustapha Mokrane

## Main Text

(PATTER *1*, 100058-1–10; August 14, 2020)

Due to an error in production, at time of publication the references were incorrectly ordered, and as such, the citations were linked to the incorrect reference. Also, the corresponding author email was incorrect and should have been updated from hylke.koers@surfsara.nl to hylke.koers@surf.nl. These errors have now been corrected online.

