# Supplementary material for: Recommendations for Services in a FAIR Data Ecosystem
Source: Patterns (N Y). 2020 Jul 7;1(5):100058. doi: 10.1016/j.patter.2020.100058 (PMC7660419; doi:10.1016/j.patter.2020.100058)
Supplement: Document S1. Table S1 [file mmc1.pdf]

PATTER, Volume *1*

Supplemental Information

Recommendations for Services in a FAIR Data Ecosystem

Hylke Koers, Daniel Bangert, Emilie Hermans, René van Horik, Maaike de Jong, and Mustapha Mokrane

Certification	TFiR Recommendation	Comment	Category	Step	Type of recommendation
Certification mechanisms and capability maturity models need to be further developed for and embraced by services to align with FAIR Principles	Rec 13: Develop metrics to certify FAIR services		Incentives & metrics for FAIR data and servic	3: Embed & sustain	Priority
Data repositories should undergo FAIR aligned certification such as CoreTrustSeal	Rec 9: Certify FAIR services		FAIR ecosystem	2: Implement	Priority
**Essential infrastructure components**					
PID services for a wide range of objects, such as publications, researchers, data sets and organisations. Emerging PID types (e.g. for instruments) should be monitored and used when they are mature	Rec 3: Develop components of a FAIR ecosystem	The recommendation presented here is more specific in the sense that it focused on PID services and types	Concepts for FAIR implementation	1: Define	Priority
Domain-specific ontologies, as domain-specific requirements have to be taken into account	Rec 7: Support semantic technologies		FAIR ecosystem	2: Implement	Priority
Human and machine-readable standards to make datasets findable, reusable and interoperable (licences as one particular example of standards needed for machine readability)	Rec 3: Develop components of a FAIR ecosystem		Concepts for FAIR implementation	2: Implement	Priority
If applicable, metadata that complies with appropriate (domain) standards should be generated and captured automatically (for e.g by instruments)	Rec 8: Facilitate automated processing	The recommendation presented here is more specific in the sense that it focuses on automatic metadata creation at data capture	FAIR ecosystem	2: Implement	Priority
**Stewardship**					
Establish data stewardship programmes providing simple and intuitive training for researchers, and enable data stewards and researchers who support applications of FAIR	Rec 10: Professionalize data science & stewardship roles & Rec 11: Implement curriculum frameworks and training		Skills for FAIR	2: Implement	Priority
Support preservation and appraisal of research outputs: Improve and maintain FAIRness of data objects over time and the long-term usability and findability of datasets	Rec 19: Select and prioritize FAIR digital objects		FAIR culture	2: Implement	Supporting
**Costs**					
Determine the cost for services to align with FAIR principles including for data management support, maintenance and long-term preservation	Rec 18: Cost data management	The recommendation presented here focuses more strongly on cost determination.	FAIR culture	2: Implement	Supporting
Develop a sustainable funding model (of services) taking into account that there might be additional costs for FAIR	Rec 14: Provide strategic and coordinated funding & Rec 15: Provide sustainable funding		Investment in FAIR	3: Embed & sustain	Priority
Provide support when determining the cost of data management as this is typically underestimated or unknown	Rec 18: Cost data management	The recommendation presented here focuses more strongly on cost determination.	FAIR culture	2: Implement	Supporting
**Rewards**					
Consider FAIR compliance and data sharing as part of research assessment, among other criteria	Rec 6: Recognize & reward FAIR data & stewardship		FAIR culture	2: Implement	Priority
References to use certified Trustworthy Digital Repositories (TDRs) in Data Management Plans should be recognised and recommended by funders	Rec 20: Deposit in Trusted Digital Repositories	The recommendation presented here is more specific in the sense that it specifically suggests that TDR's should be referenced in DMP's	FAIR culture	2: Implement	Supporting
**Collaboration and support**					
Set-up and participate in cross-institutional, collaborative communities of practice to advance and implement FAIR services	Rec 23: Develop components to meet research need	The recommendation presented here focuses more strongly on the value of community building in itself	FAIR ecosystem	2: Implement	Supporting
Foster global collaboration on FAIR implementation challenges and emerging solutions through organisations such as the Research Data Alliance	n/a	New element: foster global collaboration			
Create practical guidelines on how to enable FAIR in repositories	Rec 16: Apply FAIR broadly	The recommendation presented here is more specific in that it focuses on data repositories.	Concepts for FAIR implementation	1: Define	Supporting
Provide skilled legal advisers in institutions to help in preparing robust DMPs	Rec 5: Ensure data management via DMP's	The recommendation presented here is more specific in that it calls specifically for legal advisers.	FAIR culture	2: Implement	Priority
**Data management**					
There should be a data selection policy that – pre-deposit – recognises that not all research outputs must meet the highest levels of FAIRness, and recognizes· what has long term value, and has effect immediately after generation	Rec 19: Select and prioritize FAIR digital objects	The recommendation presented here considers levels of FAIRness, whereas the TFiR recommendation appears to be more binary ("what to keep and make FAIR and what not")	FAIR culture	2: Implement	Supporting
Data Management Plans should be required early when applying for funding and must have organisational relevance	Rec 5: Ensure data management via DMP's		FAIR culture	2: Implement	Priority
Legal aspects should be taken into account from the start of a project	n/a	New element: focus on including legal aspects at the start of a project			

Table S1: Mapping of the recommendations presented here onto those from TFiR.
