# Supplementary material for: Recommendations for Services in a FAIR Data Ecosystem
Source: Patterns (N Y). 2020 Jul 7;1(5):100058. doi: 10.1016/j.patter.2020.100058 (PMC7660419; doi:10.1016/j.patter.2020.100058)
Supplement: Document S2. Article plus Supplemental Information [file mmc2.pdf]

Patterns

CellPress
OPEN ACCESS

Perspective

Recommendations for Services in a FAIR Data Ecosystem

Hylke Koers,[1,6,*] Daniel Bangert,[2,6] Emilie Hermans,[3,6] René van Horik,[4,6] Maaike de Jong,[5,6] and Mustapha Mokrane[4,6]
[1]SURFsara, 1098 XG Amsterdam, the Netherlands
[2]Göttingen State and University Library, University of Göttingen, 37073 Göttingen, Germany
[3]Ghent University, 9000 Ghent, Belgium
[4]DANS-KNAW, 2593 HW The Hague, the Netherlands
[5]Royal Netherlands Academy of Arts and Sciences, 1011 JV Amsterdam, the Netherlands
[6]These authors contributed equally
*Correspondence: hylke.koers@surf.nl
https://doi.org/10.1016/j.patter.2020.100058

THE BIGGER PICTURE This article puts forward recommendations for data and infrastructure service providers to support findable, accessible, interoperable, and reusable (FAIR) research data within the scholarly ecosystem. Formulating such recommendations is important to coordinate progress in realizing a FAIR data ecosystem in which research data can be easily shared and optimally reused, with the aim of driving down inefficiencies in the current academic system and enabling new forms of data-driven discovery. Key recommendations—ranked by their perceived urgency—resulting from an extensive community consultation process include that (1) funders and institutions should consider FAIR alignment and data sharing as part of research assessment, among other criteria; (2) services should support domain-specific ontologies by identifying disciplines that lack ontologies and enriching existing registries of ontologies; (3) repositories should support FAIR data by developing tools, such as APIs, sharing best practices, and undergoing FAIR-aligned certification; and (4) institutions should support FAIR awareness and implementation by establishing data stewardship programs providing simple and intuitive training for researchers. The recommendations outlined in this article are meant to help guide the way forward to putting into practice the FAIR guiding principles for data management.

1 2 3 4 5 **Proof-of-Concept:** Data science output has been formulated, implemented, and tested for one domain/problem

The development and growing adoption of the FAIR data principles and associated standards as a part of research policies and practices place novel demands on research data services. This article highlights common challenges and priorities and proposes a set of recommendations on how data infrastructures can evolve and collaborate to provide services that support the implementation of the FAIR data principles, in particular in the context of building the European Open Science Cloud (EOSC). The recommendations cover a broad area of topics, including certification, infrastructure components, stewardship, costs, rewards, collaboration, training, support, and data management. These recommendations were prioritized according to their perceived urgency by different stakeholder groups and associated with actions as well as suggested action owners. This article is the output of three workshops organized by the projects FAIRsFAIR, RDA Europe, OpenAIRE, EOSC-hub, and FREYA designed to explore, discuss, and formulate recommendations among stakeholders in the scientific community. While the results are a work-in-progress, the challenges and priorities outlined provide a detailed and unique overview of current issues seen as crucial by the community that can sharpen and improve the roadmap toward a FAIR data ecosystem.

Introduction

Some 6 years after their formulation, the FAIR guiding principles for scientific data management and stewardship[1,2] have fueled the development of a wide array of policies, standards, practices, and technology in support of better research data management.[3] Also, today, the principles underpin ongoing work in developing the European Open Science Cloud[4–6] (EOSC) and shaping a "FAIR ecosystem" as envisioned in the Turning FAIR into Reality (TFiR) Report published in 2018.[7] The report defines the FAIR ecosystem as "A model proposed in the current report

This is an open access article under the CC BY license (http://creativecommons.org/licenses/by/4.0/).

denoting the minimal components needed to offer an ecosystem that enables the creation, curation, and reuse of FAIR Digital Objects in an effective and sustainable way'' and argues how such an ecosystem is essential to realize FAIR, i.e., to make research data truly findable, accessible, interoperable, and reusable on a global scale.

Central to the FAIR data ecosystem as proposed in TFiR is the notion of a FAIR Digital Object, which is illustrated in Figure 1. These FAIR Digital Objects are accompanied by data services, registries, and interoperability standards—and the system is underpinned by metrics, certification mechanisms, incentives, funding, and training for FAIR data skills. TFiR proposes a set of 27 Recommendations divided over 6 main categories (concepts, culture, ecosystem, skills, incentives and metrics, investment) to support the realization of a FAIR data ecosystem. These are currently being elaborated in forums and working groups, such as the EOSC Working Groups (WGs) on FAIR and Architecture. In particular, the EOSC FAIR WG has recently published interim recommendations on service certification[8] and metrics,[9] including initial ideas collected at the EOSC Symposium (2019, Budapest) about which services should be certified and which criteria should guide decisions around establishing formal certification vis-a-vis more informal sharing of good practices. The initial recommendations of the EOSC FAIR WG concerning metrics are to include disciplinary diversity and to consider FAIR and metrics as a continuous process that is to be evaluated and updated based on set guidelines. For the recommendations on service certifications the focus is on the work of the FAIRsFAIR project and to gather input on certifying repositories and other services based on 2 of the priority recommendations of the TFiR: one from the FAIR ecosystem chapter, ''Develop assessment frameworks to certify FAIR services'' (Rec. 9), and one from the Incentives and Metrics chapter, ''Develop metrics to certify FAIR services'' (Rec. 13).

This article aims to improve and sharpen the TFiR recommendations and associated roadmap, by rooting them in the needs of the research community, analyzing gaps and identifying areas for improvement. Focusing specifically on the role of data services in a FAIR data ecosystem, this article brings together and presents what the community perceives to be the most urgent challenges and opportunities for services to support FAIR data. This is a timely effort because there has been a lot of activity on further developing and specifying the concept of ''FAIR data'' in general and within specific academic communities—often in the form of FAIR assessment tools[10,11]—but it is much less clear what should be expected from a data service in the FAIR data ecosystem.

This topic of ''FAIR services'' (i.e., services supporting the production and management of FAIR Digital Objects) rose to prominence at the EOSC summit in 2017 and has enjoyed considerable interest since. As mentioned above, TFiR specifically includes recommendations aimed at services and signals that ''more work is needed to extend the FAIR data principles for application to a wide range of data services, including registries, Data Management Planning tools, metadata standards and vocabulary bodies, identifier providers, software libraries and other cloud services.'' This is not an easy feat, because a thorough application of the FAIR data principles and their implications for services should, on one hand, be rooted in actual research practices and, on the other, connect to the vision of the EOSC as an overarching system. At present, the implementation and adoption of the FAIR data principles within various research communities is still in development, while the EOSC is also a work-in-progress.

The TFiR report makes an explicit recommendation (Rec. 9 ''Certify FAIR services'') to use existing community-endorsed certification frameworks for Trustworthy Digital Repositories—in particular CoreTrustSeal—as a useful starting point to develop assessment frameworks for FAIR services. CoreTrustSeal (www.coretrustseal.org) requirements are formulated from an infrastructure-centric perspective with a focus on data preservation and organizational sustainability. They incorporate the aspects addressed by the FAIR data principles[12] and thus certified repositories through appropriate data curation and stewardship enable a baseline FAIRness level to the datasets they hold and contribute to maintain or even increase the level of FAIRness over time.

Alongside other ongoing work[13,14] that takes a more top-down approach to developing a FAIR assessment framework for services, the present article contributes to the discussion by capturing and presenting input from various stakeholder groups regarding their needs and expectations from services in a FAIR data ecosystem.

Based on 3 workshops organized by the projects FAIRsFAIR, Research Data Alliance (RDA) Europe, OpenAIRE, EOSC-hub, and FREYA, this article examines challenges and identifies priorities for how services and infrastructures can help researchers meet the FAIR data principles for their research data. The intent of the 3 workshops was to gather recommendations from the community on how to turn the vision of FAIR data and supporting services into reality through community consultation.

The outcomes gathered in these workshops are aimed at a variety of action owners: research services and infrastructures (who intend to integrate alignment with FAIR data principles into the architecture of their systems) but also funders, institutions, universities, and organizations, such as the EOSC and the RDA. This is especially relevant with the development and implementation of the EOSC.

The initial 2 workshops examined services in the research data ecosystem and discussed challenges and recommendations for services to support FAIR data through panels and breakout groups. During these 2 workshops, key needs and areas for improvement were identified by participants. The most notable areas for improvement were a lack of a sustainable ecosystem of independent interoperable services; findability and accessibility, which requires mostly technical expertise and specific domain expertise for increasing interoperability and reuse; and skills and services for data stewardship and preservation.

After the community consultations yielded a list of recommendations, the third workshop set out to prioritize those recommendations, define actions to be taken, and suggest stakeholders best suited for taking responsibility to carry these actions forward. While the prioritization exercise showed substantial heterogeneity between different stakeholder groups, essential infrastructure components and socially oriented recommendations around fostering global collaborations and including FAIR in research assessments scored high among all groups.

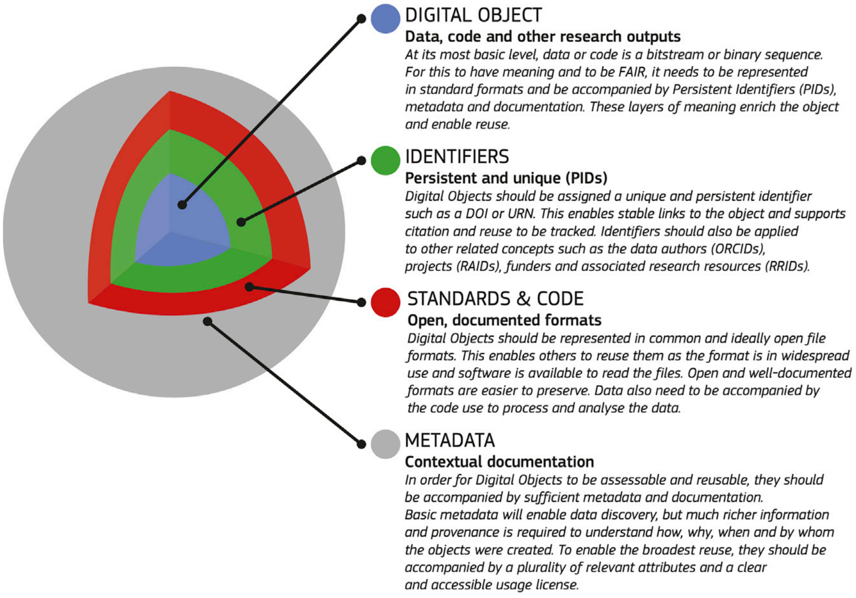

Figure 1. A Model for FAIR Digital Objects, which Lies at the Heart of the Notion of a FAIR Ecosystem as Proposed in the "Turning FAIR into Reality" Report
Figure reused from this report.[7]

These priorities and actions provide further direction and impetus to the development of a FAIR data ecosystem, in particular in the context of building the EOSC. Furthermore, cooperation between stakeholders, an opportunity provided by these workshops, is necessary to build a holistic ecosystem.

Gathering Recommendations from the Community

The workshop series was organized as 3 half-day events held in April and September 2019. The workshops examined services in the scholarly and research data ecosystem: what exists, what could be modified, and how can service provisioning be optimized. These events also provided an opportunity to engage with experts and a range of stakeholders on how to turn the vision of FAIR data and services into reality.

The first workshop (Prague, April 2019) was targeted at service providers and research infrastructures. At this workshop, 3 implementation stories were presented on services and initiatives to help make data FAIR, such as the certification of data repositories, services for data management and exploitation, and persistent identifier (PID) services. In breakout groups, workshop participants then discussed challenges and recommendations concerning services to support FAIR data.

The primary audience of the second workshop (Vienna, April 2019) consisted of research support staff and researchers. Four implementation stories were presented, followed by breakout groups and a panel discussion. The objectives of this workshop were to share perspectives on how to assist researchers with applying the FAIR data principles, to explore existing services and extensions needed to support FAIR research outputs, to understand how services can work together, and to identify further recommendations for supporting FAIR data.

The services presented at these events offered a sample representing the minimum components of the FAIR data technical ecosystem identified in TFiR. The presentations and discussions covered the broader scholarly ecosystem, recognizing that FAIR data are part of a complex and evolving landscape.

Gaps

During the 2 workshops, key needs and areas of improvement were identified by participants. Within the current research data management landscape, some of the biggest gaps include:

1. Lack of a sustainable ecosystem of independent interoperable services with governance, business model(s), and shared responsibilities to support the creation of FAIR research outputs.
2. With respect to the functionality of services the following should be addressed equally: (1) the principles related to findability and accessibility, which require mostly technical expertise that can be addressed by generic services (e.g., PIDs, cataloging, discovery and storage) and (2) the principles related to interoperability and reuse, which require services that cater to disciplinary needs with specific domain expertise (e.g., ontologies, curation, and stewardship provided by domain repositories).
3. Skills and services for data stewardship and preservation are needed to maintain the FAIRness of research outputs over time. Technical and conceptual expertise for data services is necessary.

Recommendations

Suggestions from the first 2 workshops resulted in an initial set of recommendations for services to support FAIR data. These are collated below, grouped into 7 broad categories:

Certification

1. Certification mechanisms and capability maturity models need to be further developed for and embraced by services to align with FAIR Principles.
2. Data repositories should undergo FAIR-aligned certification, such as CoreTrustSeal.

Essential Infrastructure Components

Services supporting FAIR data should offer or make use of the following components:

1. PID services for a wide range of objects, such as publications, researchers, datasets, and organizations. Emerging

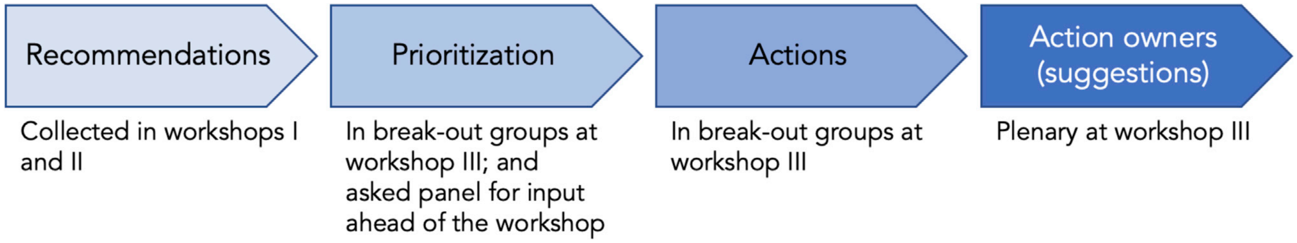

Figure 2. Approach to Prioritizing Recommendations

PID types (e.g., for instruments) should be monitored and used when they are mature.
2. Domain-specific ontologies, as domain-specific requirements have to be taken into account.
3. Human and machine-readable standards to make datasets findable, reusable and interoperable (licenses as one particular example of standards needed for machine readability).
4. If applicable, metadata that comply with appropriate (domain) standards should be generated and captured automatically (e.g., by instruments).

Stewardship

To support the effective use and uptake of services enabling FAIR, institutions should:

1. Establish data stewardship programs providing simple and intuitive training for researchers and enable data stewards and researchers who support applications of FAIR.
2. Support preservation and appraisal of research outputs: improve and maintain FAIRness of data objects over time and the long-term usability and findability of datasets.

Costs

1. Determine the cost for services to align with FAIR principles, including the costs for data management support, maintenance, and long-term preservation.
2. Develop a sustainable funding model (of services) taking into account that there might be additional costs for FAIR.
3. Provide support when determining the cost of data management as this is typically underestimated or unknown.

Rewards

1. Consider the level of FAIRness and data sharing as part of research assessment, among other criteria.
2. References to certified trustworthy digital repositories in data management plans should be recognized and recommended by funders.

Collaboration and Support

1. Set up and participate in cross-institutional, collaborative communities of practice to advance and implement services to support FAIR data.
2. Foster global collaboration on FAIR implementation challenges and emerging solutions through organizations, such as the RDA.
3. Create practical guidelines on how to enable FAIR data in repositories.
4. Provide skilled legal advisers in institutions to help in preparing robust data management plans.

Data Management

1. There should be a data selection policy that—predeposit—recognizes that not all research outputs must meet the highest levels of FAIRness, and recognizes what has long-term value, and has effect immediately after generation.
2. Data management plans should be required early when applying for funding and must have organizational relevance.
3. Legal aspects should be taken into account from the start of a project.

Prioritization of Recommendations

Following the gathering of recommendations in workshops I and II, the third and final workshop (Porto, Sept. 2019) set out to solidify the work and produce outputs to guide the community in the development of services to support FAIR data. The overall approach is illustrated in Figure 2 and may be summarized as follows:

1. Take stock of recommendations gathered so far
2. Assign relative priorities to the recommendations
3. Associate actions to the top priority recommendations
4. Collect community input on “action owners,” i.e., who could take those actions forward

This section will detail the process that was followed to prioritize the recommendations; actions and action owners is discussed in the following section.

Prioritization Process

To assign relative priorities to each recommendation, we divided the audience into 3 groups. These groups were chosen to align with different stakeholders: research institutions, service providers, and libraries. For each of these, we followed a straightforward ranking exercise: every group member received a total of 10 “votes” which they could freely distribute over the various recommendations. It must be noted that the participants were asked to indicate what should be done first (rather than what should be done versus not done), and thus low scores do not necessarily reflect that the recommendation is not of importance in the long term. Participants were free to give all their votes to a single recommendation, divide their votes over 10 recommendations, or anything in between. We then tallied the votes per

CellPress
OPEN ACCESS

		Research institutions	Service providers	Libraries	Panel
	**Certification**				
◔	Certification mechanisms and capability maturity models need to be further developed for and embraced by services to align with FAIR Principles	*	**	*	***
◑	Data repositories should undergo FAIR aligned certification such as CoreTrustSeal	***	**	***	**
	**Essential infrastructure components**				
●	PID services for a wide range of objects, such as publications, researchers, data sets and organisations. Emerging PID types (e.g. for instruments) should be monitored and used	**	***	****	****
◕	Domain-specific ontologies, as domain-specific requirements have to be taken into account	***	****	*	****
◑	Human and machine-readable standards to make datasets findable, reusable and interoperable (licences as one particular example of standards needed for machine	*	***	***	**
●	If applicable, metadata that complies with appropriate (domain) standards should be generated and captured automatically (for e.g by instruments)	****	****	**	****
	**Stewardship**				
◑	Establish data stewardship programmes providing simple and intuitive training for researchers, and enable data stewards and researchers who support applications of FAIR	****	**	**	**
◑	Support preservation and appraisal of research outputs: Improve and maintain FAIRness of data objects over time and the long-term usability and findability of datasets	**	*	****	***
	**Costs**				
◑	Determine the cost for services to align with FAIR principles including for data management support, maintenance and long-term preservation	**	*	***	***
◕	Develop a sustainable funding model (of services) taking into account that there might be additional costs for FAIR	**	***	***	***
○	Provide support when determining the cost of data management as this is typically underestimated or unknown	*	*	**	**
	**Rewards**				
●	Consider FAIR compliance and data sharing as part of research assessment, among other criteria	****	**	****	***
○	References to use certified Trustworthy Digital Repositories (TDRs) in Data Management Plans should be recognised and recommended by funders	**	*	*	**
	**Collaboration and support**				
◔	Set-up and participate in cross-institutional, collaborative communities of practice to advance and implement FAIR services	*	*	**	****
●	Foster global collaboration on FAIR implementation challenges and emerging solutions through organisations such as the Research Data Alliance	****	***	**	****
◔	Create practical guidelines on how to enable FAIR in repositories	*	****	*	*
○	Provide skilled legal advisers in institutions to help in preparing robust DMPs	*	*	**	*
	**Data management**				
◑	There should be a data selection policy that – pre-deposit – recognises that not all research outputs must meet the highest levels of FAIRness, and recognizes· what has long	*	****	****	*
◔	Data Management Plans should be required early when applying for funding and must have organisational relevance	****	**	*	*
◔	Legal aspects should be taken into account from the start of a project	*	**	***	*

Figure 3. Prioritized Recommendations

recommendations to yield a prioritization score for every recommendation (simply put, most votes meant highest priority score).

In addition to gathering input from the community, we assembled a panel of experts in the field representing different backgrounds and communities:

- Ian Duncan, Australian Research Data Commons (ARDC)
- Françoise Genova, Center de Données astronomiques de Strasbourg (CDS) and EOSC FAIR WG
- Odile Hologne, French Institute for Agricultural Research (INRA), EOSC Rules of Participation WG, and FAIRsFAIR Champion
- Rachael Kotarski, The British Library, EOSC FAIR WG, and FREYA project
- Tobias Weigel, German Climate Computing Center (DKRZ), EOSC Architecture WG, and FAIRsFAIR champion

Input from the 5 panelists was collected before the workshop in the form of a prioritized ordering of all recommendations. Scores were then assigned according to the priority order (highest score to the top priority and so on). Averaging the scores across panelists resulted in a priority score for each recommendation from the panel as a whole.

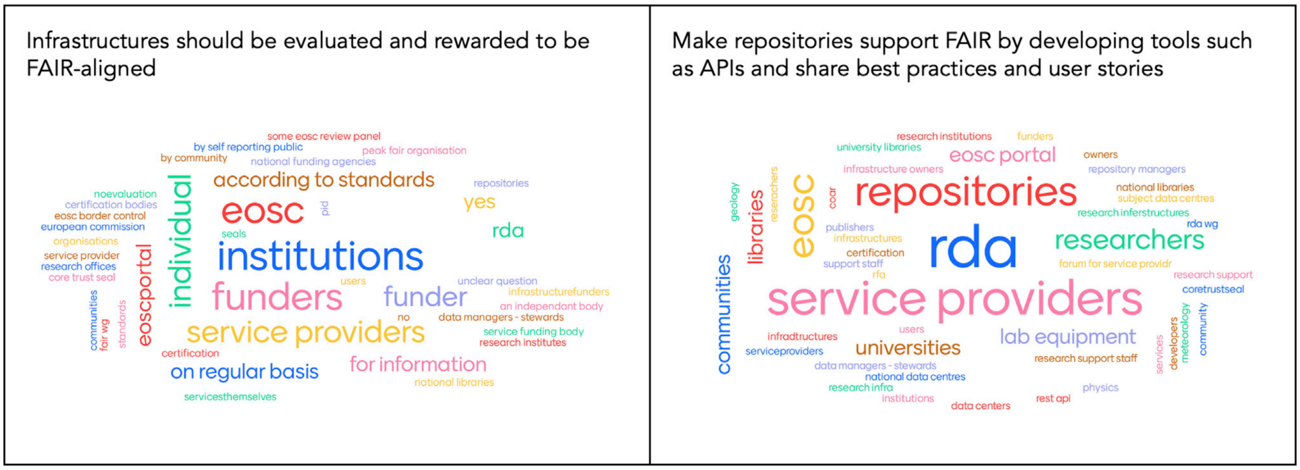

Figure 4. Audience Input in the Form of Word Clouds on Possible Action Owners for Two of the Actions Defined by the Breakout Groups

As the panel consisted of 5 people and the breakout groups consisted of 10 to 15 people each, we recognize that there will be sizable statistical fluctuations in the priority scores calculated this way. To reduce statistical noise, we aggregated data by clustering the recommendations into quartiles, meaning that we only distinguish between 4 categories:

- First quartile: low priority, denoted by 1 star (*)
- Second quartile: medium priority, denoted by 2 stars (**)
- Third quartile: high priority, denoted by 3 stars (***)
- Fourth quartile: top priority, denoted by 4 stars (****)

Any conclusions and recommendations in this article are based on these broad categories rather than on the exact priority scores.

Prioritized Recommendations

The outcome of the prioritization exercise is summarized in Figure 3. The various recommendations are displayed as rows, while the different stakeholder groups, plus the expert panel, are distributed over the columns. The color coding indicates the relative priority, from 1 star (light red) for lowest priority to 4 stars (dark green) for the highest. As explained above, the relative priority corresponds to the quartile of the overall vote distribution.

Finally, the Harvey balls on the left of Figure 3 indicate the overall ranking assigned to the recommendation when weighted equally over the different stakeholder groups plus panel (i.e., a full Harvey ball means highest overall priority).

As may readily be observed in Figure 3, there is substantial variability between priorities as assigned by the different stakeholder groups. For example, "practical guidelines on how to enable FAIR in repositories" was seen as a top priority by service providers but as a low priority by the other stakeholder groups and the panel. Similarly, "establishing data stewardship programmes" was seen as a top priority by research institutions but only as a medium priority by the others.

Notwithstanding this variability, 4 recommendations stand out as being assigned at least medium priority by all, and top priority by 2 different groups. They are the following:

- PID services for a wide range of objects, such as publications, researchers, datasets, and organizations. Emerging PID types (e.g., for instruments) should be monitored and used when they are mature.
- If applicable, metadata that complies with appropriate (domain) standards should be generated and captured automatically (e.g., by instruments).
- Consider FAIR alignment and data sharing as part of research assessment, among other criteria.
- Foster global collaboration on FAIR implementation challenges and emerging solutions through organizations, such as the RDA.

Two further recommendations with a high priority are:

- Domain-specific ontologies, as domain-specific requirements have to be taken into account.
- Develop a sustainable funding model (of services) taking into account that there might be additional costs for FAIR.

From Recommendations to Actions

With recommendations now prioritized, participants in the breakout groups brainstormed possible actions to implement their top recommendation, considering feasibility. From the actions suggested, again a top action was selected by the different stakeholder groups. It should be noted that these actions reflect the discussions in the different stakeholder groups at the time and are not necessarily suitable for generalization.

The selected priorities and subsequent actions discussed by the breakout groups formed the basis for a discussion about the stakeholders that could take on the responsibilities for various actions in the services ecosystem. To gather input from the audience on possible action owners for the identified actions, we used the interactive presentation tool Mentimeter. Figure 4 provides examples of feedback on 2 of the actions: 1 formulated by the libraries group and 1 by the service providers group.

Table 1 presents the priorities selected by the 3 stakeholder groups, the matching actions they thought to be most

Table 1. Priorities, Actions, and Suggested Action Owners according to Stakeholder Group

Group	Priority	Action	Suggested Action Owners
Libraries	Consider FAIR alignment and data sharing as part of research assessment, among other criteria.	Infrastructures should be evaluated and rewarded to be FAIR-aligned; reward researchers who apply the FAIR principles to their research, e.g., through incentives such as increased visibility for their work	EOSC, funders, service providers, community, universities
Service providers	Domain-specific ontologies, as domain-specific requirements have to be taken into account.	Identify disciplines which do not have ontologies and create awareness for registries of ontologies and enrich them[a]; make repositories support FAIR by developing tools, such as APIs, and share best practices and user stories	RDA, services providers, repositories
Research institutions	Establish data stewardship programs providing simple and intuitive training for researchers, and enable data stewards and researchers who support applications of FAIR.	Identify and present the cost of developing supporting infrastructure, including human resources	Service providers, institutions, EOSC

[a]The BARTOC registry was specifically mentioned during the breakout groups.

appropriate, and the 3 most commonly suggested action owners for each action.

The workshop concluded with an open discussion involving the expert panel and audience. A number of additional considerations were raised, such as the need for EOSC to include an overarching Authentication and Authorization Infrastructure; a suggestion to implement highly automatable Digital Object management holistically along the whole research data life cycle; and an encouragement to involve national libraries in the discussions and events around FAIR.

Discussion

Figure 3 presents an overview of the recommendations together with the relative priority assigned by different stakeholder groups and a panel of experts. In this section we discuss our interpretation of these results and relate it to previous work, in particular to the recommendations presented in TFiR.

As a first observation, the figure exhibits substantial heterogeneity, with different stakeholder groups assigning different priorities to the various recommendations (and occasionally disagreeing among themselves). We take this to reflect the relatively low level of maturity with regards to the realization of services to support FAIR data—characterized by many simultaneous challenges, limited information, or validation of "what works," and various actors reviewing or redefining their roles and responsibilities. In addition, different stakeholders will view the world through different lenses and may have different underlying priorities. For example, it is not surprising that service providers assign high priority to the essential infrastructure components but less, compared with others, to issues of data stewardship. Similarly, rewards are seen as a high priority by research institutes and libraries—who have ample experience with incentivizing researchers to make their data FAIR—but less so by service providers who are perhaps more distant to these challenges.

Notwithstanding this heterogeneity, an area that seems to stand out as a confirmed priority is that of essential infrastructure components—including services to automatically create metadata, PID services, and domain-specific ontologies. Complementary to this more technical dimension, socially oriented recommendations around fostering global collaborations and including "making data FAIR" in research assessments also scored well across the different stakeholder groups.

Naturally the various recommendations do not stand in isolation but are related, dependent, and sometimes interdependent. For example, "Improving and maintaining FAIRness of data objects over time" (category: stewardship) will be greatly helped by having in place "Practical guidelines on how to enable FAIR data in repositories" (collaboration and support), as well as "References to certified Trusted Digital Repositories in Data Management Plans should be recognized and recommended by funders" (rewards).

A detailed overview of further links and dependencies would be interesting; however, we will not pursue such an analysis here because our recommendations and categorizations are based on bottom-up community input rather than a systematic design, making them less suitable for such detailed modeling. Instead, to get a clearer view of dependencies between technical and nontechnical aspects, we relate this work to a wider body of knowledge as described in TFiR. This has the added benefit of placing our recommendation into a roadmap and elaborates the broader context of implementing FAIR.

In a similar vein to the FAIRsFAIR report on Policy Enhancement Recommendations,[15] we have mapped the 20 recommendations presented here to the 27 recommendations from TFiR (which are divided over 15 priority recommendations and 12 supporting recommendations). We found that 9 of our recommendations can be mapped rather straightforwardly to the TFiR recommendations, whereas another 9 can also be related to TFiR recommendations but add a different focus or emphasis. For example, the recommendation "Set-up and participate in cross-institutional, collaborative communities of practice to advance and implement FAIR services" is similar in spirit to TFiR Rec. 23 "Develop components to meet research needs"; however, it places more emphasis on collaboration and community building as an element of value in and of itself.

"Turning FAIR into reality" category	Certification	Essential infrastructure components	Stewardship	Costs	Rewards	Collaboration and support	Data management
Define: Concepts for FAIR implementation		2				1	
Implement: FAIR culture			1	2	2	1	2
Implement: FAIR ecosystem	1	2				1	
Implement: Skills for FAIR			1				
Embed & sustain: Incentives & metrics for FAIR data and services	1						
Embed & sustain: Investment in FAIR				1			
(not mapped)						1	1

Figure 5. Mapping of the Clustering of Recommendations Presented Here to the Categories Introduced in the "Turning FAIR into Reality" Report

We found that 2 recommendations from the present work could not be readily mapped to those from TFiR, which would suggest they contain additional suggestions or perspectives:

- "Foster global collaboration on FAIR implementation challenges and emerging solutions through organizations, such as the Research Data Alliance"
- "Legal aspects should be taken into account from the start of a project"

The first of these recommendations clearly signals how the community values international collaboration and consensus-building. To be clear, the importance of such activities is also acknowledged in TFiR, but it is not stated as a recommendation in itself. Similarly, our recommendations place somewhat greater emphasis on legal aspects and the determination of costs for data management and the cost for data services to align with the FAIR principles (although note cost management in a broader sense is included in TFiR Rec. 18: "Cost data management").

Vice versa, the following TFiR priority recommendations are not explicitly mapped to our recommendations:

- Rec. 1: "Define FAIR for implementation"
- Rec. 2: "Implement a model for FAIR Digital Objects"
- Rec. 4: "Develop interoperability frameworks"
- Rec. 12: "Develop metrics for FAIR Digital Objects"

We note that these recommendations address the architecture, information model, and interoperability frameworks underpinning the FAIR data ecosystem. Therefore, they can be seen as enablers for many of the recommendations presented here, rather than direct value providers. Therefore, it is perhaps not surprising that they were not formulated explicitly by the stakeholder groups engaged here. In the same vein, our recommendations do not explicitly call out the need for standard interfaces between various infrastructural components, even though standards and interfaces will be essential for a sustainable system. Among the nascent interfaces that could be used and further developed as part of this emerging ecosystem are Event Data (https://datacite.org/eventdata.html), which exposes links between data and the literature following the Scholix framework[16] (http://www.scholix.org/).

While the full mapping is included as supplementary material to this article (Table S1), Figure 5 presents an aggregate view of how the 7 categories introduced in this work relate to the 6 categories from TFiR. While there is some clustering around for example our category of "Essential infrastructure components" and the TFiR categories of "Concepts for FAIR implementation," and "FAIR ecosystem," most categories have more diffuse relationships. For instance, the TFiR category "FAIR culture" cuts across 5 of our 7 categories. We explain this by noting that the categories introduced in this work are derived from thematic analysis, whereas the TFiR categories are more functionally oriented and have a defined ordering in time.

With this mapping in mind, we suggest that a useful next step in operationalizing the insights and recommendations presented here would be to elaborate in more detail on how they might improve, sharpen, and add to those from TFiR and subsequently reevaluate the roadmap as laid out in TFiR.

Conclusion

This article presents the outcome of an active process of community consultation—most notably in the form of 3 workshops held in 2019—to gather, discuss, and analyze recommendations for data services and research infrastructures to support the implementation of the FAIR guiding principles for scientific data management. Coming from a broad range of participants, representing several stakeholder groups, these recommendations provide valuable insights into what are perceived to be the greatest impediments, challenges, and opportunities for services to support FAIR data. These insights give further direction and impetus to the development of a FAIR data ecosystem as envisioned in the TFiR report, in particular in the context of building the EOSC. To deliver tangible and actionable results, with a view to facilitating adoption, the recommendations gathered in the initial 2 workshops were prioritized and associated with actions and suggested action owners in the third and final workshop. Here, it should be clarified that "priority" is meant as a statement of timeliness more than overall value; in other words, participants were explicitly asked to indicate what should be done the most urgently rather than what should be done versus not done.

The recommendations and priorities are outlined in Figure 3 and analyzed in detail in the Discussion section. In brief, while we observe a fairly strong degree of heterogeneity—which we attribute to the relatively low maturity of the field as well as to the different interests and perspectives among the various stakeholder groups—there are some areas that stand out. A clear priority across the stakeholder groups is the availability of essential infrastructure components, including services to automatically create metadata, PID services, and domain-specific ontologies. Complementary to this more technical dimension, socially oriented recommendations around fostering global collaborations and including FAIR in research assessments were also prioritized across the different stakeholder groups.

We have mapped our recommendations to the recommendations presented in TFiR (see Figure 5). This mapping is intended to provide a starting point for future work to operationalize the insights and recommendations presented here and ensure that they feed into a roadmap of work ahead. In addition to this, our recommendations will be used in ongoing activities in the Horizon 2020 projects FAIRsFAIR, OpenAIRE, FREYA, EOSC-hub, and RDA Europe, as well as the EOSC FAIR Working Group and other relevant projects. In particular, FAIRsFAIR task 2.4 will benefit from these recommendations in the development of a FAIR assessment framework for services.[13]

Furthermore, it is hoped that some readers might recognize themselves as a stakeholder or action owner and find this article helpful in developing services, infrastructure, tools, ontologies, standards, models, policies, and practices that will be supported and valued by the community. Finally, as another tangible follow-up activity, we were pleased to receive requests to reuse the workshop format to gather and discuss community input in other geographical regions, which could help to corroborate findings and make progress toward an inclusive and truly global FAIR data ecosystem.

SUPPLEMENTAL INFORMATION

Supplemental Information can be found online at https://doi.org/10.1016/j.patter.2020.100058.

ACKNOWLEDGMENTS

The authors thank the speakers, panelists, moderators, and discussion group facilitators involved in the workshop series: Ian Duncan, Enol Fernandez, Françoise Genova, Natalie Harrower, Lars Holm Nielsen, Odile Hologne, Sarah Jones, Stefan Kasberger, Mari Kleemola, Rachael Kotarski, Peter Kraker, Simon Lambert, Natalia Manola, Konstantinos Repanas, Tony Ross-Hellauer, Barbara Sanchez, Andra Waagmeester, and Tobias Weigel. We also acknowledge the support from the organizing teams of EOSC-hub Week, Linking Open Science in Austria, and the Open Science Fair and thank all workshop participants for their valuable input. FAIRsFAIR "Fostering FAIR Data Practices In Europe" has received funding from the European Union's Horizon 2020 project call H2020-INFRAEOSC-2018-2020 grant agreement no. 831558. RDA Europe 4.0 has received funding from the European Union's Horizon 2020 (H2020) research and innovation program under grant agreement no. 777388. OpenAIRE-Advance has received funding from the European Union's Horizon 2020 (H2020) research and innovation program under grant agreement no. 777541. The FREYA project has received funding from the European Union's Horizon 2020 research and innovation program under grant agreement no. 777523. EOSC-hub has received funding from the European Union's Horizon 2020 research and innovation program under grant agreement no. 777536.

AUTHOR CONTRIBUTIONS

Conceptualization, H.K., D.B., E.H., R.H., and M.M.; Methodology, H.K., D.B., E.H., R.H., M.J., and M.M.; Investigation, H.K., D.B., E.H., R.H., M.J., and M.M.; Writing – Original Draft, H.K., D.B., E.H., R.H., M.J., and M.M.; Writing – Review & Editing, H.K., D.B., E.H., R.H., M.J., and M.M.; Visualization, H.K.

DECLARATION OF INTERESTS

The authors are involved in the following projects: FAIRsFAIR, RDA Europe, OpenAIRE-Advance OpenAIRE, EOSC-hub, and FREYA. Mustapha Mokrane is a member of the CoreTrustSeal Standards and Certification Board.

REFERENCES

1. The FAIR Data Principles FORCE11; 2014. https://www.force11.org/group/fairgroup/fairprinciples.
2. Wilkinson, M.D., Dumontier, M., Aalbersberg, I.J.J., Appleton, G., Axton, M., Baak, A., et al. (2016). The FAIR Guiding Principles for Scientific Data Management and Stewardship. Sci. Data *3*, 160018, https://doi.org/10.1038/sdata.2016.18.
3. Mons, B., Neylon, C., Velterop, J., Dumontier, M., da Silva Santos, L.O.B., and Wilkinson, M.D. (2017). Cloudy, increasingly FAIR; revisiting the FAIR data guiding principles for the European Open Science Cloud. Inf. Serv. Use *37*, 49–56, https://doi.org/10.3233/ISU-17084.
4. Jones, S., and Abramatic, J.-F. (2019). European Open Science Cloud (EOSC) Strategic Implementation Plan (Directorate-General for Research and Innovation (European Commission)). https://doi.org/10.2777/202370.
5. European Commission (2017). EOSC Declaration, https://ec.europa.eu/research/openscience/pdf/eosc_declaration.pdf.
6. EUDAT, LIBER, OpenAIRE, EGI, and GEANT. (2015). Position Paper: European Open Science Cloud for Research. Zenodo. https://doi.org/10.5281/zenodo.32915.
7. European Commission Expert Group on FAIR Data (2018). Turning FAIR into Reality: Final Report and Action Plan from the European Commission Expert Group on FAIR Data (Brussels: Directorate-General for Research and Innovation (European Commission)). https://doi.org/10.2777/1524.
8. Genova, F., Aronsen, J.M., Beyan, O., Harrower, N., Holl, A., McIntosh, L., Principe, P., Repanas, K., Slavec, A., and Jones, S. (2020). Interim recommendations on certifying the services required to enable FAIR research outputs within EOSC (EOSC FAIR Working Group), https://repository.eoscsecretariat.eu/index.php/s/zCnHTcytBHaLjRp#pdfviewer.
9. Genova, F., Aronsen, J.M., Beyan, O., Harrower, N., Holl, A., Hooft, R.W.W., McIntosh, L., Principe, P., Repanas, K., Slavec, A., et al. (2020). Interim recommendations on FAIR Metrics for EOSC (EOSC FAIR Working Group), https://repository.eoscsecretariat.eu/index.php/s/C3a5WkpsFHL6GD3#pdfviewer.
10. Bahim, C., Dekkers, M., and Wyns, B. (2019). Results of an Analysis of Existing FAIR Assessment Tools (Research Data Alliance). https://doi.org/10.15497/RDA00035.
11. Wilkinson, M.D., Sansone, S.-A., Schultes, E., Doorn, P., Bonino da Silva Santos, L.O., and Dumontier, M. (2018). A design framework and exemplar metrics for FAIRness. Sci. Data *5*, 1–4, https://doi.org/10.1038/sdata.2018.118.
12. Mokrane, M., and Recker, J. (2019). CoreTrustSeal-certified repositories: enabling findable, accessible, interoperable, and reusable (FAIR). In 16th International Conference on Digital Preservation (iPRES). https://doi.org/10.17605/OSF.IO/9DA2X.
13. Koers, H., Gruenpeter, M., Herterich, P., Hooft, R., Jones, S., Parland-von Essen, J., and Staiger, C. (2020). Assessment report on 'FAIRness of services'. Zenodo, https://doi.org/10.5281/zenodo.3688762.
14. L'Hours, H., and von Stein, I. (2019). FAIR Ecosystem Components: Vision (FAIRsFAIR project). https://doi.org/10.5281/zenodo.3565428.
15. Davidson, J., Grootveld, M., Whyte, A., Herterich, P., Engelhardt, C., Stoy, L., et al. (2020). D3.3 Policy Enhacement Recommendations. Zenodo. https://doi.org/10.5281/zenodo.3686901.
16. Burton, A., Koers, H., Manghi, P., Stocker, M., Fenner, M., Aryani, A., et al. (2017). The Scholix Framework for Interoperability in Data-Literature Information Exchange. D-Lib Mag *23*, https://doi.org/10.1045/january2017-burton.

About the Authors

Dr. Hylke Koers heads up the Data Management Services group at SURF, which develops services that help research institutes manage their research data. Hylke joined SURF in 2018; before that he was a Product Director at Elsevier, working on innovations to the scholarly article, such as interactive plots and the Virtual Microscope. He co-founded the ICSU-WDS/RDA Working Group that created the Scholix framework, an emerging industry standard for linking research data and the literature. He currently leads FAIRsFAIR task 2.4 on "FAIRness" of data services & software, and contributes to the EOSC architecture WG and EOSC-hub WP12. Hylke holds a Ph.D. in theoretical astrophysics from the University of Amsterdam.

Dr. Daniel Bangert is a Scientific Manager in the Knowledge Commons unit of the Göttingen State and University Library, University of Göttingen. He contributes to projects related to open science and research data management, including the Research Data Alliance (RDA) Europe and FAIRsFAIR. His

background includes roles in data librarianship, repository services, and scholarly communications. Daniel holds qualifications in information management, music performance, and musicology, including a Ph.D. in music from the University of New South Wales.

Emilie Hermans is National Open Access Desk for Belgium and involved in the coordination of the OpenAIRE network as well as in institutional support for Open Science at Ghent University. She is involved in developing learning material, providing training and support, research data management, and organizing events. Recently she is also involved in the dissemination and outreach work package of EOSC-Pillar.

Dr. René van Horik is project manager at Data Archiving and Networked Services (DANS-KNAW) in the Netherlands and involved in EU projects EOSC-hub, FREYA, SSHOC, and FAIRsFAIR, which are related to the European Open Science Cloud (EOSC). He is a member of the EOSC skills and training working group. His main activities concern research and training and outreach in the field of research data management and certification of data repositories. René holds a Ph.D. in information science from Delft University of Technology.

Dr. Maaike de Jong is policy adviser at the Netherlands Royal Academy of Arts and Sciences (KNAW). During the past 4 years she worked as project leader and researcher at KNAW institute Data Archiving and Networked Services (DANS-KNAW), where she developed and managed international projects in open science and open data. She led the engagement work package of FREYA, an EU project on persistent identifiers. Maaike has a background in biological research and holds a Ph.D. in evolutionary ecology and genetics from Leiden University.

Dr. Mustapha Mokrane is project leader at Data Archiving and Networked Services (DANS-KNAW) in the Netherlands, where he co-leads the FAIRsFAIR project funded by the European Union to assist the European Open Science Cloud (EOSC) governance bodies deliver FAIR-aligned Rules of Participation. His expertise is in research data management and policy. He was previously Executive Director of the International Science Council's World Data System. His scientific background is in genetics and bioinformatics and he holds a Ph.D. in Molecular Biology from the Aix-Marseille University.

PATTER, Volume *1*

Supplemental Information

Recommendations for Services in a FAIR Data Ecosystem

Hylke Koers, Daniel Bangert, Emilie Hermans, René van Horik, Maaike de Jong, and Mustapha Mokrane

Certification	TFiR Recommendation	Comment	Category	Step	Type of recommendation
Certification mechanisms and capability maturity models need to be further developed for and embraced by services to align with FAIR Principles	Rec 13: Develop metrics to certify FAIR services		Incentives & metrics for FAIR data and servic	3: Embed & sustain	Priority
Data repositories should undergo FAIR aligned certification such as CoreTrustSeal	Rec 9: Certify FAIR services		FAIR ecosystem	2: Implement	Priority
**Essential infrastructure components**					
PID services for a wide range of objects, such as publications, researchers, data sets and organisations. Emerging PID types (e.g. for instruments) should be monitored and used when they are mature	Rec 3: Develop components of a FAIR ecosystem	The recommendation presented here is more specific in the sense that it focused on PID services and types	Concepts for FAIR implementation	1: Define	Priority
Domain-specific ontologies, as domain-specific requirements have to be taken into account	Rec 7: Support semantic technologies		FAIR ecosystem	2: Implement	Priority
Human and machine-readable standards to make datasets findable, reusable and interoperable (licences as one particular example of standards needed for machine readability)	Rec 3: Develop components of a FAIR ecosystem		Concepts for FAIR implementation	2: Implement	Priority
If applicable, metadata that complies with appropriate (domain) standards should be generated and captured automatically (for e.g by instruments)	Rec 8: Facilitate automated processing	The recommendation presented here is more specific in the sense that it focuses on automatic metadata creation at data capture	FAIR ecosystem	2: Implement	Priority
**Stewardship**					
Establish data stewardship programmes providing simple and intuitive training for researchers, and enable data stewards and researchers who support applications of FAIR	Rec 10: Professionalize data science & stewardship roles &<br>Rec 11: Implement curriculum frameworks and training		Skills for FAIR	2: Implement	Priority
Support preservation and appraisal of research outputs: Improve and maintain FAIRness of data objects over time and the long-term usability and findability of datasets	Rec 19: Select and prioritize FAIR digital objects		FAIR culture	2: Implement	Supporting
**Costs**					
Determine the cost for services to align with FAIR principles including for data management support, maintenance and long-term preservation	Rec 18: Cost data management	The recommendation presented here focuses more strongly on cost determination.	FAIR culture	2: Implement	Supporting
Develop a sustainable funding model (of services) taking into account that there might be additional costs for FAIR	Rec 14: Provide strategic and coordinated funding &<br>Rec 15: Provide sustainable funding		Investment in FAIR	3: Embed & sustain	Priority
Provide support when determining the cost of data management as this is typically underestimated or unknown	Rec 18: Cost data management	The recommendation presented here focuses more strongly on cost determination.	FAIR culture	2: Implement	Supporting
**Rewards**					
Consider FAIR compliance and data sharing as part of research assessment, among other criteria	Rec 6: Recognize & reward FAIR data & stewardship		FAIR culture	2: Implement	Priority
References to use certified Trustworthy Digital Repositories (TDRs) in Data Management Plans should be recognised and recommended by funders	Rec 20: Deposit in Trusted Digital Repositories	The recommendation presented here is more specific in the sense that it specifically suggests that TDR's should be referenced in DMP's	FAIR culture	2: Implement	Supporting
**Collaboration and support**					
Set-up and participate in cross-institutional, collaborative communities of practice to advance and implement FAIR services	Rec 23: Develop components to meet research need	The recommendation presented here focuses more strongly on the value of community building in itself	FAIR ecosystem	2: Implement	Supporting
Foster global collaboration on FAIR implementation challenges and emerging solutions through organisations such as the Research Data Alliance	n/a	New element: foster global collaboration			
Create practical guidelines on how to enable FAIR in repositories	Rec 16: Apply FAIR broadly	The recommendation presented here is more specific in that it focuses on data repositories.	Concepts for FAIR implementation	1: Define	Supporting
Provide skilled legal advisers in institutions to help in preparing robust DMPs	Rec 5: Ensure data management via DMP's	The recommendation presented here is more specific in that it calls specifically for legal advisers.	FAIR culture	2: Implement	Priority
**Data management**					
There should be a data selection policy that – pre-deposit – recognises that not all research outputs must meet the highest levels of FAIRness, and recognizes· what has long term value, and has effect immediately after generation	Rec 19: Select and prioritize FAIR digital objects	The recommendation presented here considers levels of FAIRness, whereas the TFiR recommendation appears to be more binary ("what to keep and make FAIR and what not")	FAIR culture	2: Implement	Supporting
Data Management Plans should be required early when applying for funding and must have organisational relevance	Rec 5: Ensure data management via DMP's		FAIR culture	2: Implement	Priority
Legal aspects should be taken into account from the start of a project	n/a	New element: focus on including legal aspects at the start of a project			

Table S1: Mapping of the recommendations presented here onto those from TFiR.
